# Exploring the Associations Between Inflammatory Biomarkers, Survival, and Cardiovascular Events in Hemodialysis Patients and the Interrelationship with Nutritional Parameters—The Experience of a Single Transylvanian Dialysis Center

**DOI:** 10.3390/jcm14041139

**Published:** 2025-02-10

**Authors:** Crina Claudia Rusu, Ina Kacso, Diana Moldovan, Alina Potra, Dacian Tirinescu, Maria Ticala, Yuriy Maslyennikov, Alexandra Urs, Cosmina Ioana Bondor

**Affiliations:** 1 Department of Nephrology, University of Medicine and Pharmacy “Iuliu Hatieganu” Cluj, 8 Victor Babes, Street, 400012 Cluj-Napoca, Romania; 2 Department of Nephrology, County Emergency Clinical Hospital Cluj, 3-5 Clinicilor Street, 400006 Cluj-Napoca, Romania; 3 Department of Medical Informatics and Biostatistics, University of Medicine and Pharmacy “Iuliu Hatieganu” Cluj, 6 Pasteur Street, 400349 Cluj-Napoca, Romania

**Keywords:** kidney disease, diagnosis, biomarkers, nutrition, survival, cardiovascular events, hemodialysis

## Abstract

**Background/Objectives:** In hemodialysis (HD), inflammatory biomarkers are discussed as prognostic markers for survival and cardiovascular events (CVEs). The results of the studies are not uniform and there are particularities related to population groups and comorbidities. In addition, it is known that inflammation determines protein malnutrition and less about the effect of adipose tissue on inflammation in HD. This study investigates the relationship between inflammatory molecules and nutritional biomarkers, and CVE and survival in HD patients. **Methods:** We included, in an observational, longitudinal study, 65 patients with chronic HD (53 without diabetes and 22 smokers), with a mean age of 60.1 ± 12.4 years. High-sensitivity C-reactive protein (hs-CRP), interleukin 1 beta, tumor necrosis factor alpha (TNF-alpha), interleukin 6, soluble tumor necrosis factor-like weak inducer of apoptosis (sTWEAK), soluble CD163 (sCD163), and fibroblast growth factor 21 were determined. We recorded survival and cardiovascular events for 60 months. Univariate and multivariate analyses were performed. **Results:** Hs-CRP was significantly associated with survival (*p* = 0.014) in the total group. In smokers and former smokers, TNF-α lower than 368.34 pg/mL was associated with better survival. In multivariate analysis, hs-CRP was correlated with adipose tissue biomarkers (*p* = 0.006), and sCD163 was correlated with total and LDL cholesterol (*p* = 0.002). In addition, in univariate analysis, sTWEAK was correlated with serum albumin (*p* = 0.026, r = −0.30). **In conclusion**, in HD patients, hs-CRP was significantly associated with survival, and low TNF-alpha values in smokers and former smokers were linked to better survival. Hs-CRP was also correlated with adipose tissue biomarkers, CD163 was correlated with total and LDL cholesterol, and albumin was inversely associated with sTWEAK. The relation between inflammatory molecules and adipose tissue biomarkers was less identified in HD patients until now.

## 1. Introduction

In patients on chronic hemodialysis (HD), the elevated risk of morbidity and mortality from cardiovascular disease (CVD) can be attributed to both traditional and non-traditional CKD-specific risk factors. Among the non-traditional factors, malnutrition, inflammation, and atherosclerosis (ATS) are components of the malnutrition–inflammation–atherosclerosis (MIA) syndrome [1,2,3]. In HD patients, protein malnutrition is frequently observed and is associated with increased morbidity and mortality [4,5]. Inflammation in CKD is a protective physiological response [6], but it can also become maladaptive, uncontrolled, and persistent over time [7]. As CKD progresses, patients have an increasingly inflammatory state [8]. In chronic HD patients, inflammation can occur secondarily to dialysis membranes, central venous catheters, oxidative stress, cellular aging, hypoxia, fluid overload, sodium overload, immune dysfunction, intestinal dysbiosis, and retention of uremic toxins [9,10]. Inflammatory molecules are produced by immunocompetent cells and cardiomyocytes, endothelial cells, adipocytes, and vascular smooth muscle cells [11,12]. Numerous inflammatory markers have been evaluated for their impact on CV pathology and survival in patients with chronic HD, including molecules such as high-sensitivity C-reactive protein (hs-CRP), tumor necrosis factor alpha (TNF-α), and interleukin 6 (IL-6). In contrast, interleukin 1 beta (IL-1β), the weak inducer of apoptosis similar to soluble tumor necrosis factor (sTWEAK), soluble CD163 (sCD163), and fibroblast growth factor 21 (FGF 21) have been less studied. IL-1β, IL-6, TNF-α, and hs-CRP are hallmarks of low-grade inflammation in CKD [13]. TNF-α regulates adipose tissue metabolism [12,14], and IL-6 stimulates the hepatic synthesis of acute-phase proteins, including CRP [15]. sTWEAK, a member of the TNF family, circulates in plasma as a soluble form [16], can induce smooth muscle cell proliferation in the arterial wall [17], and is involved in all pathogenetic phases of ATS [18]. Like sTWEAK, CD163 is a transmembrane protein with a soluble variant that can be measured in serum [19]. sTWEAK can bind and block sCD163 [20]. sCD163 levels are increased in obese and hypertensive patients [21,22] and are directly correlated to the severity of ATS in CKD [21,22]. The liver and adipose tissue secrete fibroblast growth factor 21 (FGF21), an endocrine-like cytokine [23]. Elevated FGF21 levels have been associated with the development of major acute cardiovascular events [24,25]. The cytokines in HD patients can predict survival rates and cardiovascular events. However, the predictive ability of different competing molecules has been less frequently evaluated [9,26,27,28,29,30] and may vary depending on population characteristics and comorbidities. In addition, a few studies focusing on hemodialysis patients have examined the relationship between inflammatory mediators and nutritional status in a dual manner: which mediators are related to adipose tissue and which are associated with muscle mass. Furthermore, exploring the correlations between different inflammatory molecules may provide insights into the pathogenetic cascades involved in CVD in HD patients.

This study aims to evaluate the relationship between inflammatory molecules (hs-CRP, IL-6, TNF-α, IL-1β, sCD163, sTWEAK, and FGF21) and cardiovascular events and survival in chronic hemodialysis patients within a specific sample from a single center. In addition, we will examine the relationship between these inflammatory mediators, including novel ones, and nutritional status, assessed by anthropometric and laboratory parameters. We will also explore the connections between different inflammatory molecules.

## 2. Materials and Methods

We performed a comprehensive observational study on a cohort of HD patients randomly selected from a chronic dialysis center in Cluj-Napoca. We included patients with prevalent HD and a minimum age of 18 years, with at least 6 months of HD maintenance treatment (HD duration), and without residual renal function (thus, residual renal function cannot be discussed as a factor influencing nutrition, respectively, in the inflammation markers of the studied patients). Exclusion criteria were acute inflammation, terminal neoplasia, previous renal transplantation, immunosuppressive treatment, and active hepatitis. We registered demographic data, HD duration, and comorbidities (diabetes, hypertension, CVD, hepatitis B or C infection, smoking status, and medication) from their medical records. Additionally, we recorded clinical data: age, weight, height, systolic blood pressure (SBP) and diastolic blood pressure (DBP) (pre-dialysis values), history of CVD, waist circumference (WC) (cm), and triceps skinfold thickness (TST) (mm). Pulse pressure was calculated with the following formula: (PP): PP = SBP − DBP (mmHg).

To evaluate body mass index (BMI), we used the formula BMI = weight (kg)/height^2^ (m^2^). The other anthropometric parameters were measured by bioimpedance using the Body Composition Monitor, a certified device (manufactured by Fresenius Medical Care, Bad Homburg, Germany) that provided body composition as follows: lean tissue mass (LTM) (kg) and adipose tissue mass (ATM) (kg) [31]. 

We followed patients prospectively for 60 months or until death or transplantation. Throughout this time, we documented general mortality, fatal cardiovascular events (myocardial infarction, congestive heart failure, stroke, and sudden death), and nonfatal cardiovascular events every six months. According to other authors, the term stroke can be replaced by cerebrovascular insult [32]. We calculated the survival time (ST) as the interval between the study entry and death and the time to a cardiovascular event (TCVE) as the interval between the study entry and the first recorded cardiovascular event.

### 2.1. Laboratory Parameters

All biochemical analyses were performed after an overnight fast between 7.00 and 9.00 a.m., always during a midweek non-dialysis day. Current measurements at the initiation of this study include serum electrolytes, albumin, creatinine, uric acid, iron profile (iron, transferrin, and ferritin), lipid profile (total cholesterol, triglycerides (TGs), and HDL cholesterol), hs-CRP, alkaline phosphatase, intact parathormone (iPTH), and transaminases. Pre-dialysis and post-dialysis urea levels were used to calculate Kt/V. Serum calcium was corrected (cCa) for albumin according to the following formula: cCa (mg/dL) = serum calcium (mg/dL) + 0.8 × [4.0-serum albumin (g/dL)]. Hepatitis virus B and C detection was performed by electrochemiluminescence for HBs antigen (HBs Ag) and hepatitis C virus antibodies (HCV Ab). We already determined Serum IL-6, IL-1β, TNF-α, sTWEAK, sCD163, and FGF21 with an enzyme-linked immunosorbent assay (ELISA) using commercially available kits (R&D System, Minneapolis, MN, USA). The minimum detection limit for TNF-α was 15.6 pg/mL, for IL-6—3.2 pg/mL, for IL-1β—10.2 pg/mL, and for sTWEAK—10 pg/mL, and the intra- and inter-assay coefficients of variation were 7.9% and 9.1%, respectively; for sCD163, the minimum detectable level was 0.613 ng/mL, and the intra- and inter-assay coefficients of variation were 5.1 and 3.5%, respectively, while for FGF21, the detection limit was 7 pg/mL and the intra- and inter-assay coefficients of variation were 8 and 8.7%.

### 2.2. Dialysis Prescription

All included patients received conventional HD treatment three times a week, 4–5 h per session (only 3 patients had 5 h/session, the rest, 4 h/session), and were dialyzed with disposable synthetic dialyzers (polysulfone) and heparin as a standard anticoagulant. A nephrologist guided the dialysis prescription, achieving a Kt/V value ≥ 1.4. Erythropoietin was administered according to a standardized algorithm. Antihypertensive treatment was indicated for persistent post-dialysis or inter-dialysis blood pressure > 150/90 mmHg, and ultrafiltration during HD was performed based on dry weight.

### 2.3. Statistical Analysis

Numerical characteristics were summarized as mean ± standard deviation or median (25th–75th percentile) according to the normal and non-normal distribution, and qualitative characteristics were expressed as numbers and percentages. Pearson’s or Spearman correlation coefficient was used for analysis if correlations between two continuous variables were present. The inflammatory markers were analyzed in the multivariate regression models adjusted for age and dialysis duration for the total group. Collinearity was evaluated using the Variance Inflation Factor (VIF). For those variables with high VIFs, a dimension reduction was made with factor analysis. The resulting composite variables were introduced in the multivariate linear model as an independent variable.

The univariate Cox proportional hazards regression analysis examined the relationships between different independent factors and survival time without an event. For the inflammatory markers that significantly impacted survival time in Cox proportional hazards regression analysis, we also evaluated their effect on survival using Kaplan–Meier analysis. The quantitative variables were transformed into dichotomous variables using a rounded cut-off value. The cut-off values were found with the receiving operating characteristics curve, using the maximum sensitivity and specificity. The calculation of cumulative survival probabilities was plotted using the Kaplan–Meier method, and the curves were compared using the log-rank test. Significant variables significant in the univariate analysis were included in a multivariate Cox proportional hazards regression model (Enter). The models were adjusted for age and duration of dialysis. The hazard ratio (HR) and their 95% confidence intervals (CIs) were calculated. Separate multivariate Cox proportional hazards regression models were analyzed with all the inflammatory markers as independent variables.

For the analysis in this study, SPSS version 25.0 was used [33]

### 2.4. Ethical Issues

All patients provided written informed consent. The study was conducted according to the Declaration of Helsinki and approved by the Ethics Committee of “Iuliu Hatieganu” University of Medicine and Pharmacy Cluj-Napoca 348/26 September 2017.

## 3. Results

### 3.1. Total Group Description

We enrolled 65 randomly selected patients who met the inclusion and exclusion criteria. Two (3.1%) patients were excluded due to missing data regarding their transfer to other hospitals; 63 remained in the study, 53 did not have diabetes, and 22 were current and former smokers. The mean follow-up period was 51.81 ± 15.77 months. The number of patients who died in the follow-up period was 17 [26.2%; 95% CI (16; 37.9)], and 24 [36.9%; 95% CI (26.1; 50)] patients had CVE.

The clinical and laboratory patients’ characteristics at the time of inclusion in the study are shown in Table 1. In the study, we had a small number of patients with diabetes mellitus (15.4%), with an average age of 60.78 ± 11.68 years, 63% male.

### 3.2. Correlations Between Inflammatory Markers and Nutritional and Lipid Parameters

The correlations between inflammatory markers and nutritional and lipid parameters are presented in Table 2.

We found significant correlations between the following inflammatory mediators: hs-CRP with sCD163, sTWEAK with FGF 21, sTWEAK with TNF-α, and TNF-α with IL-6 (Table 2). In addition, we found significant correlations between the following inflammatory mediators and markers of lipid metabolism: WBC with triglycerides, sCD163 with triglycerides, FGF21 with triglycerides, sCD163 with Total cholesterol, and sCD163 with LDL cholesterol (Table 2).

Of the total group, in multivariate linear regression, hs-CRP remained significantly statistically correlated with WBC total (*p* = 0.034) and with the composite variable from BMI and ATM (*p* = 0.006) after adjusting with age and diabetes duration. WBC remained significantly statistically correlated with hs-CRP (*p* < 0.001) and TG (*p* = 0.035). When sCD163 was analyzed in multivariate linear regression, LDL cholesterol and total cholesterol were highly associated and were reduced to a composite variable, which remained statistically significantly associated with sCD163 in multivariate regression (*p* = 0.002). TNF-α remained significantly statistically correlated with IL-6 (*p* = 0.005).

### 3.3. Survival Time and Time to the First CVE on Total Group

Table 3 presents the effect of inflammatory molecules on survival time and on time to a cardiovascular event (TCVE) resulting from the univariate Cox proportional hazards regression analysis. Diabetes mellitus influences the time to CVE (TCVE); hs-CRP, iPTH, and calcium were other factors affecting survival time. Diastolic blood pressure was a protective factor that influenced time without CVE.

We analyzed the independent factors’ relationship with *survival time* in a multivariate Cox proportional hazards regression analysis. All the variables significant in the univariate Cox proportional hazards regression analysis, calcium, IPTH, and hs-CRP, were taken in the model and adjusted with age, kt/v, and dialysis duration. The hs-CRP [HR = 1.48, 95% CI (1.0, 1.99), *p* = 0.010] and calcium [HR = 2.83, 95% CI (1.30, 6.16), *p* = 0.009] remained significant in predicting survival time. For the *time to CVE*, the variables that remained significant in the model were diabetes mellitus [HR = 4.47, 95% CI (1.19, 16.72), *p* = 0.026], smoking (current and former) [HR = 4.88, 95% CI (1.80, 13.23), *p* = 0.002], and DBP [HR = 0.94, 95% CI (0.89, 0.98), *p* = 0.010].

**Figure 1:**
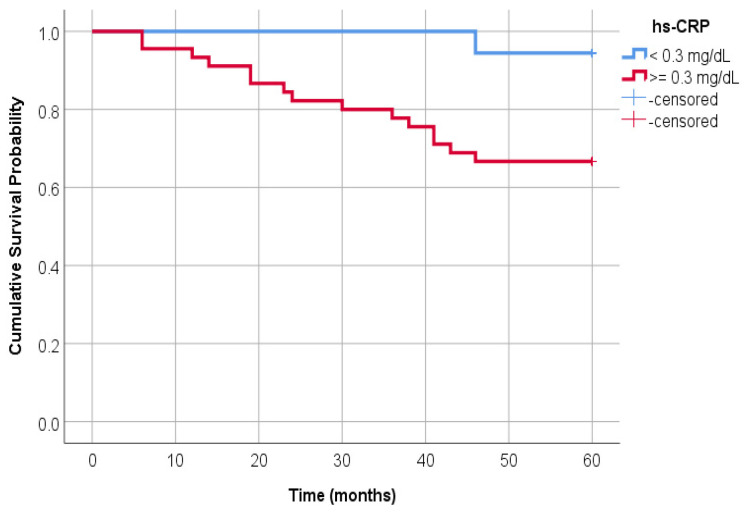
Comparative survival curves between subjects with hs-CRP less than 0.3 mg/dL and those with hs-CRP greater than or equal to 0.3 mg/dL.

The survival time was significantly different in patients with hs-CRP < 0.3 mg/dL compared to those with hs-CRP ≥ 0.3 mg/dL (Figure 1) (*p* = 0.024). In 18 patients with hs-CRP < 0.3 mg/dL, only one (5.56%) had the event (death) compared to 15 (33.3%) events in 45 patients with hs-CRP ≥ 0.3 mg/dL.

### 3.4. Survival Time and Time to the First CVE on Subgroups with and Without Diabetes

#### 3.4.1. Survival Time on Subgroups with and Without Diabetes

Univariate survival analysis in the *subgroup of diabetes* patients (n = 10) was not performed due to a small sample size.

Univariate survival analysis in the *subgroup of those without diabetes* (n = 53) revealed statistically significant factors in predicting the total survival time as follows: LTM HR = 0.91, 95% CI (0.82, 0.998), *p* = 0.045; iPTH HR = 1.001, 95% CI (1.00, 1.001), *p* = 0.026; calcium HR = 2.98, 95% CI (1.38, 6.42), *p* = 0.005; near statistically significant hs-CRP HR = 1.48, 95% CI (0.95, 2.31), *p* = 0.081; SBP HR = 0.98, 95% CI (0.95, 1.004), *p* = 0.088; and PP HR = 0.97, 95% CI (0.93, 4.00), *p* = 0.077. In multivariate analysis, after adjusting for age and dialysis duration, LTM HR = 0.87, 95% CI (0.75, 0.996), *p* = 0.026; and iPTH (HR = 1.001, 95% CI (1.00, 1.002), *p* = 0.012 remained statistically significant.

#### 3.4.2. CVE-Free Survival Time on Subgroups with and Without Diabetes

Univariate survival analysis in the subgroup of those *without diabetes* (n = 53) revealed statistically significant factors in predicting CVE-free survival time as follows: sTWEAK HR = 1.0002, 95% CI (1.00002, 1.0005), *p* = 0.029; smoking (current and former) HR = 3.21, 95% CI (1.26, 8.20), *p* = 0.015; near statistically significant serum albumin HR = 0.14, 95% CI (0.02, 1.24), *p* = 0.077; and DBP HR = 0.96, 95% CI (0.91, 1.00), *p* = 0.060. In multivariate analysis, both of the following remained statistically significant: sTWEAK HR = 1.0002, 95% CI (1.00004, 1.0005), *p* = 0.021; and smoking (current and former) HR = 2.99, 95% CI (1.17, 7.63), *p* = 0.022, but after adjusting with the age and dialysis duration only the smoking remained significant.

We did not find a significant statistical cut-off for sTWEAK in this group.

**Figure 2:**
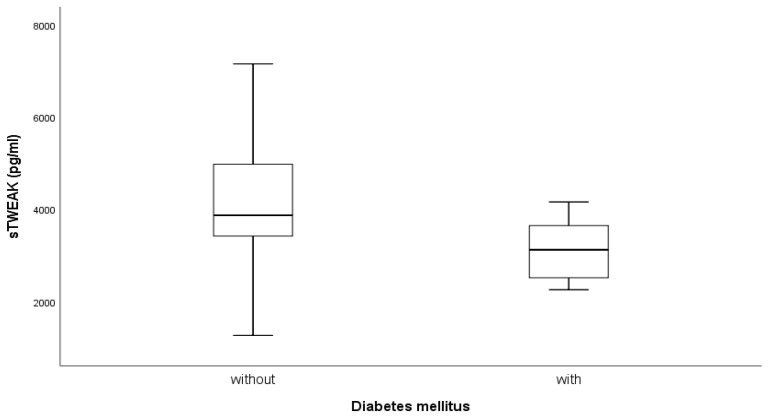
Comparative values of sTWEAK between subjects with diabetes mellitus and without.

sTWEAK was significantly different between patients with and without diabetes (*p* = 0.014) (Figure 2). STWEAK was lower in patients with diabetes.

### 3.5. Survival Time and Time to the First CVE on Subgroups of Smokers, Former Smokers, and Non-Smokers

#### 3.5.1. Survival Time on Subgroups of Smokers, Former Smokers, and Non-Smokers

Univariate survival analysis in the subgroup of smokers and former smokers (n = 22) revealed statistically significant factors in predicting total survival time as follows: TNF-α HR = 1.02, 95% CI (1.003, 1.04), *p* = 0.026; sCD163 HR = 1.001, 95% CI (1.00, 1.003), *p* = 0.036; and hs-CRP HR = 1.40, 95% CI (1.05, 1.87), *p* = 0.022. These results had a very small power ≤ 0.1. Multivariate analysis was not performed due to a small sample size.

**Figure 3:**
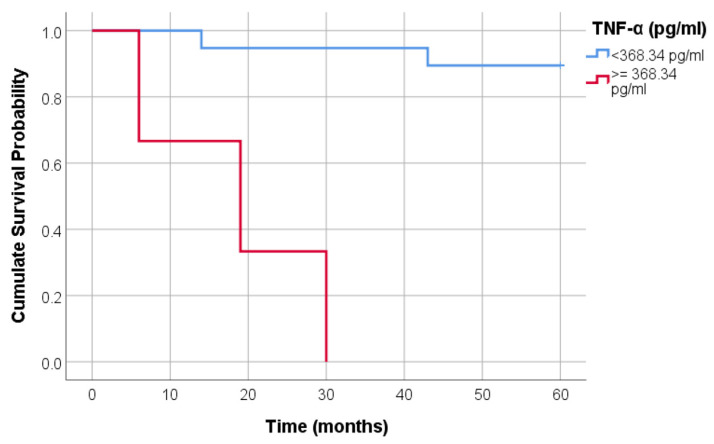
Comparative survival curves between subjects with TNF-α less than 368.34 pg/mL and those with TNF-α higher or equal to 368.34 pg/mL.

The survival time was significantly different between the smokers and former smokers with TNF-α less than 368.34 pg/mL and those with TNF-α higher or equal to 368.34 pg/mL (Figure 3) (*p* < 0.001). In nineteen patients with TNF-α < 368.34 pg/mL, only two (10.52%) had the event (death) compared to three (100%) events in three patients with TNF-α ≥ 368.34 pg/mL.

We did not find a significant statistical cut-off for sCD163 in this group.

Univariate survival analysis in the non-smoking subgroup (n = 41) shows that no inflammatory mediators influenced survival time and cardiovascular events.

#### 3.5.2. CVE-Free Survival Time on Subgroups of Smokers, Former Smokers, and Non-Smokers

Univariate survival analysis in the subgroup of smokers and former smokers (n = 22) did not highlight statistically significant factors in predicting CVE-free survival time.

Univariate survival analysis in the non-smoking subgroup (n = 41) did not highlight statistically significant inflammatory mediators predicting CVE-free survival time.

## 4. Discussion

This study showed that CRP was associated with survival in our HD patients. In addition, patients with hs-CRP levels of less than 0.3 mg/dL in the entire hemodialysis group and TNF-alpha levels of less than 368.34 pg/mL in smokers had a survival advantage in our study. Hs-CRP was correlated with adipose tissue biomarkers, a relationship that has been less defined in HD patients until now.

Hs-CRP is an inflammatory marker that is easily monitored in clinical practice. Similarly to our study, it negatively influences survival in the general population [34] and CKD, including ESRD patients [27,35,36]. In addition, hs-CRP can predict cardiovascular events in HD patients and is superior to pentraxin 3 in this prediction [35]. However, not all studies have the same results on this issue; there is still debate about which inflammatory marker better predicts cardiovascular mortality or cardiovascular events in HD patients. Thus, in one review, pentraxin 3 was associated with CVE and mortality in people with CKD [37]. IL-6 alone, or with hs-CRP, has also been associated with all-cause and cardiovascular mortality in other studies, and it was a superior predictor of malnutrition compared with other cytokines in ESRD [11,28,38,39]. In another analysis of ESRD patients, hs-CRP predicted malnutrition better than IL-6 [40]. Elevated levels of TNF-α and IL-1β have also been associated with reduced survival in chronic HD patients [41], and TNF-α has been associated with left ventricular hypertrophy in these patients [42]. Our study found that TNF alpha levels less than 368.34 pg./mL in HD smoker patients were associated with better survival. SCD163 values, too, in the same group of patients, influenced survival. It is known that smoking significantly increases all-cause mortality in dialysis patients [43], and our results indicate that one possible mechanism is the increased inflammatory process. The differences between the results of studies on inflammatory markers predictive of survival and CVE are probably due to the regional characteristics of each hemodialysis group related to lifestyle and dietary habits that influence the intestinal microbiota and, consequently, the inflammatory process. There were also differences in mean age between studies (in one review, the age range was between 45 and 87.4 years [44], in the percentage of DM, which was 15% in our study and other research [39] and around 50% in some studies [35,38], and even in the number of smokers among patients. Finally, in our opinion, based on our results and the views of other authors [35,36,39,44,45,46], CRP still proves to be an optimal and cheaper option for monitoring the inflammatory state in ESRD patients with good prognostic capacity. CRP is an acute-phase protein synthesized in hepatocytes and released in response to IL-6, IL1-β, and IL-17, secondary to signals associated with tissue damage [47,48]. The first phase is a slow release of CRP (basal levels) as the CRP pre-form, pCRP, is stored in intracellular vesicles and is converted in acute insults to the active form, mCRP [49]. In our study, we did not find correlations between hs-CRP and IL-6 or IL-1β. Other authors have also observed that in CKD (pre-dialysis and dialysis stage), there is not always a correlation between hs-CRP and IL-6 or IL-1β [50,51,52]. This may be because, besides hepatic synthesis of CRP in response to IL-6, other factors, such as genetic factors and dialysis-related factors (immune activation), can affect CRP levels [51,53]. However, we have noted that elevated hs-CRP is associated with elevated sCD163, a monocyte/macrophage-derived biomarker that reflects macrophage activation during inflammation [54]. The concentration of sCD163 in blood [40] is associated with acute and chronic inflammatory processes, fat metabolism, and CVD [55]. Regarding the role of CRP, there have been conflicting reports, and both pro- and anti-inflammatory and pro- and anti-thrombotic activities have been described [56]. CRP has only remained a diagnostic inflammatory marker because the mechanism of action is uncertain. Among patients receiving statins in the general population, high-sensitivity CRP was a stronger predictor of the risk of future cardiovascular events and death than LDL cholesterol [57].

In our study, diabetes, although present in a small percentage of patients with all associated metabolic disorders, significantly influenced CVE, as in other studies [35,58]; no inflammatory marker significantly influenced CVE in the whole group. In the subgroup analysis, we noted that although sTWEAK and smoking in nondiabetic patients were associated with CVE-free survival time in univariate and multivariate analysis, only smoking remained significantly related to CVE-free survival time after adjusting for age and duration of dialysis. More extensive studies in nondiabetic HD patients are needed to re-examine the relationship between sTWEAK and CVE. In patients undergoing chronic hemodialysis, lower levels of sTWEAK have been associated with higher carotid intima-media thickness [59] and vascular muscle dysfunction [60]. sTWEAK values decrease as CKD progresses and are particularly low in patients on hemodialysis [61]. Serum levels of sTWEAK depend on two factors: Fn 14 (a highly inducible cell surface receptor) [30] and sCD163 (a scavenger for sTWEAK) [62].

Inflammatory mediators had a dual relationship with nutritional markers in our study. We observed that high adipose mass (expressed by BMI, adipose tissue mass, waist circumference, and triceps skinfold thickness) was associated with high hs-CRP, WBC, and sCD163. In addition, in multivariate analysis, even after adjustment, hs-CRP remained related to markers of adipose tissue. This relation has been less remarked upon in other studies in HD [63]. Adipocytes are now being analyzed as the epicenter of a global pandemic of metabolic diseases [64]. White adipose tissue, the most abundant type of fat in humans, is located subcutaneously in the viscera and bone marrow. It contains dysfunctional adipocytes that secrete inflammatory cytokines and other cell types, including macrophage-like immune cells [63]. These cells produce substances that act in a paracrine and endocrine manner, affecting local and systemic metabolic responses [65,66]. Macrophage infiltration into adipose tissue in CKD has been reported to be independent of BMI [67,68,69].

Adipocytes involved in the onset of the inflammatory syndrome are also regulators of lipolysis and lipogenesis, which explains the correlations between inflammatory mediators and markers of lipid metabolism [70]. Accordingly, our study found high triglycerides associated with high sCD163 and high FGF21, resulting in an atherogenic metabolic profile. Total cholesterol and LDL cholesterol among dialysis patients also relate to nutritional status, not only to atherogenic risk. High total cholesterol levels have been associated with increased survival in chronic HD patients as an expression of the reverse epidemiology phenomenon [71]. In addition, survival in chronic HD patients is related to low inflammation [27]. In our research, we identified associations between low levels of sCD163 (low inflammation expression) and high levels of total cholesterol and LDL cholesterol in the total group (values related to good nutritional status); the association was interpreted in the same phenomenon of reverse epidemiology of cardiovascular risk in HD patients.

In addition to direct correlations between inflammatory and adipose tissue markers, many studies have shown that inflammatory markers are inversely associated with protein nutritional markers in HD patients [72,73]. This association suggests that inflammation favors protein malnutrition or, conversely, malnutrition favors the inflammatory process, both detrimental pathways for HD patients. Among HD patients, high hs-CRP has been observed to be associated with lower albumin levels [9,26], but not in our study. The absence of correlations between serum albumin and hs-CRP in our research most likely reflects a specific characteristic of the population sample studied, in which hs-CRP correlates predominantly with adipose tissue markers. However, we observed high values of other inflammatory markers, such as sTWEAK, to be associated with low albumin. We also found other markers of protein malnutrition related to inflammatory markers in subgroup analysis. In nondiabetic patients, high IL-1β was associated with low albumin, and in diabetic patients, with low lean tissue mass. In the smoking subgroup, high WBC was related to low pre-dialysis creatinine. Creatinine levels in HD patients predominantly reflect muscle mass, not renal function. Low pre-dialysis creatinine levels, as markers of protein malnutrition in chronic HD, predicted mortality [35,36,37,38,39,40]. Similarly to our results, other studies have described the relationship between inflammation and protein nutritional markers. Kaysen et al., in the HEMO study, found that CRP levels were associated with serum albumin and creatinine concentrations [74]. In addition, Johansen et al. [75] showed that during a year of longitudinal observation of 54 HD patients, CRP influenced albumin change and IL-1β modulated the change in phase angle over time. All these relationships support the malnutrition–inflammation–atherosclerosis syndrome, and this favors CVD. The pathogenesis of ATS involves an inflammatory process; in HD patients, there is a microinflammatory state. The systemic inflammatory response can increase the expression of soluble intracellular adhesion molecules and vascular endothelial growth factors [76,77] and alter blood lipids, vascular endothelium, and plasma protein composition. Lipoprotein composition and adhesion molecule changes trigger vascular injury [78,79,80,81].

The correlations between inflammatory molecules may suggest some pathogenic links and possible therapeutic targets. Above, we presented the association between CRP and sCD163. Among the other correlations, we remarked that high levels of FGF21 are associated with high levels of sTWEAK; these, in turn, are associated with high levels of TNF-α. FGF21 is associated with sTWEAK among chronic dialysis patients, which was also observed in another study by us [82]. One explanation is that, in CKD patients, elevated FGF21 levels may be secondary to a state of FGF21 resistance and have been observed to be related to the dysregulation of FGF21 receptor signaling [83]. It has been postulated that inflammation may be one of the culprits [84,85]. On the other hand, FGF21 needs βKlotho as a cofactor for its activity. sTWEAK and TNF-α reduce Klotho expression in various tissues and cells, including adipocytes, by activating NFkB and, secondarily, favor reduced FGF21 activity [86]. In turn, reduced FGF21 activity may increase compensatory synthesis. Klotho is related to FGF 23, which can exacerbate the inflammatory state [87] and promote CV disease. Also, in our study, increased levels of TNF-α were associated with increased levels of IL-6, an association explained by the pathogenic cascade of the hepatic synthesis of inflammatory cytokines [12,14]. Therefore, inflammatory molecules interact and are progressively activated in CKD, which explains their impact on survival and CVD and may raise questions about which inflammatory markers can be potential therapeutic targets.

Our research has certain limitations.

First, the small sample size may have produced bias or instability in the results. It should be noted that examining specific subgroups still leads to an even smaller sample size, increasing the likelihood that statistically significant results are due to chance. Studies with small sample sizes, like this one, face significant challenges related to statistical robustness and the reliability of results. Secondly, all participants were from a single hemodialysis center; thus, the generalizability of findings may be restricted. Lastly, the simple analysis of correlations between parameters precludes causality. Given its exploratory nature and methodological limitations, the study’s findings should be interpreted cautiously. Based on the findings of this study, future studies should be designed to explore the causal relationships between inflammation and nutrition in HD patients to facilitate better-targeted treatment.

Although it has some limitations, this study is valuable because it analyzes seven diagnostic inflammatory biomarkers and highlights the association of hs-CRP with survival in our hemodialysis (HD) patients. In addition, identifying inflammatory biomarkers associated with survival and cardiovascular events in different population groups and according to comorbidities is essential for personalizing treatments for hemodialysis patients. Our study also suggests a dual relationship between inflammation and nutrition: adipose tissue markers are associated with inflammation. At the same time, protein nutritional markers are linked to inflammation in HD patients. The relationship between inflammatory molecules and adipose tissue biomarkers has been less defined in chronic HD patients; most research has highlighted protein malnutrition associated with inflammation. Finally, although our sample size is small, the 60-month follow-up period enhances the significance and robustness of our findings.

## 5. Conclusions

CRP, a classic inflammatory biomarker, was significantly associated with survival among our HD patients. In addition, low TNF- α was associated with better survival in smokers and former smokers among HD patients. In contrast, newer biomarkers, such as IL-6, IL-1β, sCD163, sTWEAK, and FGF 21, did not show a similar influence. CRP was correlated with adipose tissue biomarkers, CD163 was correlated with total and LDL cholesterol, and albumin was inversely associated with sTWEAK. The correlation of adipose tissue and protein nutritional biomarkers with inflammatory biomarkers suggests a complex, dual relationship between inflammation and nutrition in hemodialysis patients. The relation between inflammatory molecules and adipose tissue biomarkers has been less defined in HD patients. Our findings are based on HD patients from a specific single-center sample, and extensive, multicentric studies are necessary for confirmation.

## Figures and Tables

**Table 1:** Baseline characteristics of hemodialysis patients.

Parameter	Total Group (n = 63)
Age (years)	60.78 ± 11.68
Male, n (%)	36 (63)
Current and former smokers, n (%)	22 (34.9)
Diabetes mellitus, n (%)	10 (15.4)
Hypertension, n (%)	40 (63.5)
SBP (mmHg)	142.27 ± 19.99
DBP (mmHg)	76 (70; 80)
PP (mmHg)	67.19 ± 17.37
Body mass index (kg/m^2^)	27.98 (23.94; 30.93)
Waist circumference (cm)	97.81 ± 14.56
Triceps skinfold thickness (mm)	3 (2.5; 4)
LTM (kg)	30.66 (25.52; 36.26)
ATM (kg)	41.64 ± 16.3
Dialysis duration (months)	68 (29.5; 90.5)
Kt/V	1.56 (1.39; 1.74)
LDL-cholesterol (mg/dL)	100.57 ± 38.21
Total cholesterol (mg/dL)	169 (149.5; 196.5)
HDL-cholesterol (mg/dL)	41 (31.6; 48.34)
Fasting glucose (mg/dL)	94 (88.1; 113)
Corrected Calcium (mg/dL)	8.77 (8.29; 9.18)
Phosphorus (mg/dL)	4.87 (4.09; 5.93)
Alkaline phosphatase (UI/L)	74.5 (54.78; 94.44)
iPTH (pg/mL)	300 (166.85; 789.75)
Serum albumin (g/L)	3.93 ± 0.24
Hemoglobin (g/dL)	11.39 ± 1.12
White blood cells (n/mm^3^)	6330 (5415; 7315)
hs-C reactive protein (mg/dL)	0.61 (0.25; 1.23)
TNF-α (pg/mL)	288.53 (241.91; 359.83)
IL-6 (pg/mL)	275.04 (234.58; 348.34)
IL-1β (pg/mL)	48.29 (12.72; 252.31)
FGF 21 (pg/mL)	23.34 (19.75; 37.62)
sTWEAK (pg/mL)	3738.59 (3346.57; 4721.51)
sCD163 (ng/mL)	1070 (620; 1500)

Mean ± standard deviation; median (25th–75th percentile); SBP—systolic blood pressure, DBP—diastolic blood pressure, PP—pulse pressure; LTM—lean tissue mass; ATM—adipose tissue mass; iPTH—intact parathormone; hs-C reactive protein—high-sensitivity C reactive protein; TNF-α—tumor necrosis factor alfa; IL-6—interleukin 6; IL-1β—interleukin 1 beta; FGF 21—fibroblast growth factor 21; sTWEAK—soluble tumor necrosis factor-like weak inducer of apoptosis; sCD163—soluble CD163.

**Table 2:** Correlations between inflammatory biomarkers and nutritional and lipid parameters in the study group and subgroups divided by gender, smoking status, and diabetes.

Inflammatory Marker	Nutritional/ Inflammatory/ Lipid Markers	Spearman/Pearson Coefficient of Correlation r	*p*
hs-CRP (total group)	ATM	0.34	0.007
hs-CRP (nondiabetic group)	ATM	0.31	0.026
hs-CRP (nonsmoking subgroup)	ATM	0.4	0.004
hs-CRP (nonsmoking subgroup)	WC	0.33	0.016
hs-CRP (total group)	BMI	0.26	0.046
hs-CRP (diabetic subgroup)	BMI	0.64	0.048
WBC (total group)	ATM	0.29	0.021
WBC (nondiabetic group)	ATM	0.29	0.041
WBC (total group)	WC	0.26	0.043
WBC (smoking subgroup)	Pre-dialysis creatinine	0.84	0.002
sCD163 (total group)	TST	0.48	0.048
sCD163 (nonsmoking subgroup)	TST	0.34	0.023
sTWEAK (total group)	Serum albumin	−0.30	0.026
sTWEAK (nondiabetic group)	Serum albumin	−0.39	0.007
sTWEAK (smoking subgroup)	WC	−0.68	0.043
IL-1β (diabetic subgroup)	LTM	−0.75	0.03
IL-1β (nondiabetic group)	Serum albumin	0.032	0.02
FGF21 (nonsmoking subgroup)	LTM	0.34	0.023
hs-CRP (total group)	sCD163	0.45	0.001
hs-CRP (total group)	WBC	0.55	<0.001
sTWEAK (total group)	FGF 21	0.37	0.006
sTWEAK (total group)	TNF-α	0.35	0.01
TNF-α (total group)	IL-6	0.35	0.004
WBC (total group)	Triglycerides	0.26	0.037
sCD163 (total group)	Triglycerides	0.28	0.038
sCD163 (total group)	Total Cholesterol	−0.31	0.022
sCD163 (total group)	LDL-cholesterol	−0.43	0.001
FGF 21 (total group)	Triglycerides	0.29	0.035

LTM—lean tissue mass; ATM—adipose tissue mass; TST—Triceps skinfold thickness; BMI—body mass index; WC—waist circumference; WBC—white blood cells; hs-CRP—high-sensitivity C reactive protein; TNF-α—tumor necrosis factor alfa; IL-6—interleukin 6; IL-1β—interleukin 1 beta; FGF 21—fibroblast growth factor 21; sTWEAK—soluble tumor necrosis factor-like weak inducer of apoptosis; sCD163—soluble CD163.

**Table 3:** Factors influencing survival time and time to the first CVE (TCVE).

Parameter	Total Survival Time (n = 63)		TCVE (n = 63)	
	HR (95% CI)	*p*	HR (95% CI)	*p*
Dialysis duration (months)	1.002 (0.99; 1.01)	0.646	1 (0.99; 1.01)	0.733
Age (years)	1.02 (0.98; 1.07)	0.307	1.03 (0.99; 1.07)	0.136
Male	1.27 (0.46; 3.48)	0.649	0.85 (0.38; 1.91)	0.699
Current and former smokers	0.87 (0.30; 2.49)	0.789	2.40 (1.07; 5.36)	**0.033**
Diabetes mellitus	1.81 (0.58; 5.62)	0.303	3.13 (1.23; 7.96)	**0.016**
Hypertension	1.36 (0.47; 3.92)	0.569	2.38 (0.94; 6)	0.067
SBP (mmHg)	0.99 (0.97; 1.01)	0.355	1 (0.98; 1.03)	0.703
DBP (mmHg)	1.01 (0.96; 1.06)	0.791	0.97 (0.92; 1)	**0.039**
PP (mmHg)	0.98 (0.95; 1.01)	0.230	1.02 (1; 1.05)	0.091
Body mass index (kg/m^2^)	0.94 (0.85; 1.05)	0.269	1.03 (0.96; 1.105)	0.395
LTM (kg)	0.96 (0.89; 1.03)	0.244	1 (0.95; 1.05)	0.962
Waist circumference (cm)	0.98 (0.95; 1.01)	0.222	1.01 (0.98; 1.03)	0.732
Triceps skinfold thickness (mm)	0.77 (0.49; 1.19)	0.240	1.01 (0.75; 1.36)	0.961
ATM (kg)	0.99 (0.97; 1.03)	0.888	1.01 (0.99; 1.04)	0.346
Kt/V	0.9 (0.19; 4.19)	0.895	0.91 (0.26; 3.19)	0.888
Total cholesterol (mg/dL)	0.99 (0.98; 1.01)	0.210	0.99 (0.98; 1)	0.193
LDL cholesterol (mg/dL)	0.99 (0.98; 1.00)	0.149	0.99 (0.98; 1)	0.113
HDL cholesterol (mg/dL)	1.01 (0.99; 1.04)	0.435	0.98 (0.94; 1.01)	0.217
Triglycerides (mg/dL)	1 (0.99; 1.00)	0.379	1 (1;1.01)	0.191
Fasting glucose (mg/dL)	1.003 (0.99;1.02)	0.599	1.01 (1; 1.02)	**0.043**
Calcium (mg/dL)	2.69 (1.36; 5.33)	**0.004**	0.94 (0.50; 1.80)	0.86
Phosphorus (mg/dL)	1.18 (0.90; 1.57)	0.236	1.13 (0.9; 1.43)	0.292
iPTH (pg/mL)	1.001 (1.0001;1.001)	**0.027**	1 (1; 1)	0.142
Alkaline phosphatase (UI/L)	1 (0.99; 1.01)	0.788	1 (1; 1)	0.791
Hemoglobin (g/dL)	1.14 (0.72; 1.78)	0.580	1.01 (0.68; 1.49)	0.969
Serum albumin (g/L)	0.58 (0.07; 4.34)	0.586	0.25 (0.04; 1.02)	0.12
hs-C reactive protein (mg/dL)	1.36 (1.06; 1.73)	**0.014**	1.18 (0.9; 1.56)	0.239
White blood cells (n/mm^3^)	1.0002 (1; 1.001)	0.141	1 (1; 1)	0.174
TNF-α (pg/mL)	1 (1; 1.003)	0.669	1 (1; 1)	0.884
IL-6 (pg/mL)	1 (0.99; 1.003)	0.872	1 (1; 1)	0.947
IL-1β (pg/mL)	1 (1; 1)	0.195	1 (1; 1)	0.684
FGF 21 (pg/mL)	0.99 (0.956; 1.03)	0.496	1 (1; 1)	0.482
sTWEAK (pg/mL)	1 (1; 1)	0.830	1.21 (0.28; 0.53)	0.176
sCD163 (ng/mL)	1 (1; 1.001)	0.660	1 (1; 1)	0.414

TCVE—time to cardiovascular event; HR—hazard ratio; CI—confidence interval; SBP—systolic blood pressure, DBP—diastolic blood pressure, PP—pulse pressure; LTM—lean tissue mass; ATM—adipose tissue mass; iPTH—intact parathormone; hs-C reactive protein—high-sensitivity C reactive protein; TNF-α—tumor necrosis factor alfa; IL-6—interleukin 6; IL-1β—interleukin 1 beta; FGF 21—fibroblast growth factor 21; sTWEAK—soluble tumor necrosis factor-like weak inducer of apoptosis; sCD163—soluble CD163; *p*-value—bold for factors significantly associated with survival or cardiovascular events in HD patients.

## Data Availability

The research data supporting this study’s findings are not publicly available. Further inquiries can be directed to the corresponding author.
